# Effects of Ramadan intermittent fasting on performance, physiological responses, and bioenergetic pathway contributions during repeated sprint exercise

**DOI:** 10.3389/fnut.2024.1322128

**Published:** 2024-02-09

**Authors:** Serhat Özbay, Süleyman Ulupınar, Cebrail Gençoğlu, Ibrahim Ouergui, Furkan Öget, Hasan Hüseyin Yılmaz, Necip Fazıl Kishalı, Fatih Kıyıcı, Selim Asan, İzzet Uçan, Luca Paolo Ardigò

**Affiliations:** ^1^Faculty of Sports Sciences, Erzurum Technical University, Erzurum, Türkiye; ^2^High Institute of Sport and Physical Education of Kef, University of Jendouba, El Kef, Tunisia; ^3^Research Unit, Sports Science, Health and Movement, University of Jendouba, El Kef, Tunisia; ^4^Faculty of Sports Sciences, Atatürk University, Erzurum, Türkiye; ^5^Department of Teacher Education, NLA University College, Oslo, Norway

**Keywords:** bioenergetics, Ramadan, fasting, repeated sprints, intermittent exercise

## Abstract

**Introduction:**

This investigation aims to elucidate the impact of Ramadan intermittent fasting on performance, physiological responses, and bioenergetic pathway contributions during repeated sprints.

**Methods:**

Fourteen active male Muslim athletes (age = 22.4 ± 1.8 years, body weight = 69.5 ± 3.8 kg, height = 176 ± 5.1 cm) executed a repeated sprint protocol, consisting of ten 20-meter sprints with 15-s passive recovery intervals, during both fasting and non-fasting conditions. The fasting session was conducted after a 12–14 h fast following Sahur (the pre-dawn meal during Ramadan). In contrast, the non-fasting session occurred before the Ramadan fasting period began, during the same hours of the day, at a time when fasting was not yet required for the athletes. Bioenergetic pathway contributions during repeated sprints were quantified using the PCr-LA-O_2_ method.

**Results:**

The mean sprint time during fasting sessions was 3.4 ± 0.3 s compared to 3.3 ± 0.2 s in non-fasting sessions, indicating a trend approaching the threshold of significance for slower times in the fasted state (*p* = 0.052, effect size (ES) = 0.34). In terms of bioenergetic contributions, the total metabolic energy expenditure (TEE) was slightly lower during fasting sessions (236.5 ± 22 kJ) compared to non-fasting sessions (245.2 ± 21.7 kJ), but this difference was not statistically significant (*p* = 0.102, ES = 0.40). Similarly, metabolic energy expenditure per sprint was 23.7 ± 2.2 kJ in fasting conditions compared to 24.5 ± 2.2 kJ in non-fasting conditions (*p* = 0.106, ES = 0.35). The oxidative energy contribution did not differ significantly between fasting (34.2 ± 4.1 kJ) and non-fasting conditions (34.2 ± 4.1 vs. 35.5 ± 5.2 kJ; *p* = 0.238, ES = 0.28). Similarly, lactic (60.4 ± 7.6 vs. 59.2 ± 8.3 kJ; *p* = 0.484, ES = 0.15); and alactic (149.3 ± 19.9 vs. 143 ± 21.5 kJ; *p* = 0.137, ES = 0.30) energy contributions showed no significant differences between the fasting and non-fasting sessions. The percentage of performance decrement (Pdec) and the percentage contributions of oxidative, lactic, and alactic pathways to the total energy expenditure did not differ significantly between the fasting and non-fasting conditions, indicating a similar bioenergetic profile across both conditions.

**Conclusion:**

The present findings indicate no significant differences in performance metrics and metabolic outcomes between fasted and non-fasted states. Future assessments with longer duration and higher intensity protocols may provide further insights.

## 1 Introduction

As commonly known, for religious beliefs, Muslim athletes must observe fasting from dawn to dusk during the month of Ramadan as per the Islamic lunar calendar (1, 2). During this time, both food and fluid intake are restricted during daylight hours leading to impaired muscle function, diminished metabolic energy consumption, and compromised hydration status (3–5). The daily fast during the Ramadan month is variable, dependent on the latitude and seasonal variation, but commonly lasts 10 to 20 h daily across ~30 consecutive days (6). This religious observance often conflicts with athletes’ training schedules and competitive events, as mainstream sporting calendars do not accommodate religious practices (1, 7). Ramadan intermittent fasting (RIF) has been shown to induce alterations in energy metabolism, hormonal fluctuations, and sleep pattern disruptions, which all could potentially influence athletic performances (8, 9). Although current evidence predominantly suggests that RIF may impair aerobic rather than anaerobic capacity, there is a conspicuous absence of research examining its effects on repeated sprint performance — a form of exercise that relies on both anaerobic and aerobic metabolic pathways (10, 11).

Although it is reported that maintaining adequate nutrition, sleep, and training can mitigate performance decrements during fasting (12), prolonged fasting is also associated with an inevitable decline in performance, highlighting the complex interplay between fasting duration and athletic outcomes (4). Notably, a significant gap in existing research is the insufficient focus on energy metabolism markers and other variables that could influence this process (13–15). Surveys among football players revealed that 39% of them abstain from fasting on match days and 81.5% believe that fasting could impair performance (7). These findings indicate that athletes’ decisions to fast during training or competition are complex and multifaceted, deeply rooted in personal, cultural, and religious beliefs, and not solely based on empirical evidence. This complexity underscores the importance of considering both the physiological and psychosocial dimensions of fasting in sports settings.

A critical lacuna in the existing literature is the lack of comprehensive studies examining the specific effects of Ramadan intermittent fasting (RIF) on repeated sprint activities (RSA), particularly in relation to bioenergetic pathways activation. While RSA is recognized as a key performance metric in many sports, especially in team-based games like soccer (14, 15), its interaction with the physiological and bioenergetic challenges posed by RIF remains underexplored. Given the demanding nature of RSA (16–18), involving high-intensity bursts of energy and rapid recovery, understanding how RIF influences these aspects is crucial. The lactate system, which reflects anaerobic metabolism, is particularly relevant for sports requiring high-intensity efforts, such as repeated sprints in soccer (10, 17, 19). During periods of intense physical exertion, the production of blood lactate increases significantly, serving as a marker for anaerobic metabolism and a predictor for athletic endurance and performance (20, 21). In the context of Ramadan, the fasting practice significantly alters metabolic responses and energy utilization patterns (1, 3). Fasting can lead to changes in glycogen stores, impacting lactate production and clearance (5, 22). Understanding the bioenergetic alterations during Ramadan, particularly in terms of lactate dynamics, is crucial for comprehending how fasting affects athletes’ performance during repeated sprints (23). However, the impact of fasting, particularly during Ramadan, on these high-intensity activities is not well-documented, leaving a gap in strategies for athlete preparation and training during fasting periods.

Previous research has often attributed the decline in physical performance during Ramadan to factors such as caloric restriction, altered macronutrient composition, and sleep deprivation (2, 12, 24). These elements are undoubtedly significant, yet there remains a critical gap in understanding the specific impacts of these factors on athletes’ bioenergetic pathways during Ramadan. Our investigation, therefore, takes a novel approach by controlling for these variables and focusing on shifts in energy metabolism. By isolating the influence of energy metabolism, we aim to provide a deeper understanding of how Ramadan fasting specifically affects athletic performance. This approach allows us to distinguish the effects of altered nutritional and sleep patterns from the intrinsic metabolic changes induced by fasting. Our study is not just about observing performance decrements during Ramadan but about comprehensively understanding the underlying bioenergetic mechanisms. This focus on bioenergetics is crucial as it addresses a largely unexplored aspect of sports performance during Ramadan. We hypothesize that RIF could potentially impair RSA performance by altering bioenergetic pathway contributions, thereby affecting the overall energy availability and utilization during high-intensity efforts. By investigating these dynamics, we aim to contribute valuable insights into the planning and execution of training and competitive strategies for athletes who observe Ramadan fasting. Our study’s findings could also inform approaches to optimize performance and energy management in sports disciplines where RSA is critical, extending beyond soccer to other team sports.

## 2 Materials and methods

### 2.1 Study design

This study utilized a quasi-experimental, crossover design with repeated measures to investigate the effects of RIF on RSA in active male Muslim athletes. The design involved assessing the same group of athletes under two different conditions: during Ramadan (fasting) and outside of Ramadan (non-fasting). The primary objective was to assess the impact of RIF on performance, physiological responses, and bioenergetic pathway contributions during RSA. This approach allowed for a direct comparison of each athlete’s performance under fasting and non-fasting conditions, thereby providing insights into the specific effects of Ramadan fasting on RSA performance.

### 2.2 Participants

*A priori* power analysis was calculated using the G*Power software (Version 3.1.9.4, University of Kiel, Kiel, Germany) to determine the required sample size with α set at 0.05 and power (1-β) set at 0.80. The analysis revealed that a total sample size of 13 subjects would be sufficient to find significant differences with an actual power of 0.82. Fourteen active male Muslim athletes (mean ± SD: age = 22.4 ± 1.8 years, body mass = 69.5 ± 3.8 kg, height = 176 ± 5.1 cm) voluntarily enrolled in this study. All participants were briefed on the potential risks and benefits and provided informed written consent. The study was conducted in accordance with the ethical principles outlined in the Declaration of Helsinki and received approval from the Ethics Committee of Atatürk University’s Faculty of Sport Sciences. The athletes were classified as “Trained/Developmental” based on the Participant Classification Framework by McKay et al. (25). This categorization was determined by their training frequency (3–5 times per week) and affiliation with amateur soccer teams. These athletes typically represent a performance level that includes structured training but not at the elite or professional tiers. Inclusion criteria mandated that participants be in good health, free from acute or chronic medical conditions and not undergoing any medical treatment. Exclusion criteria encompassed contraindications to maximal effort testing, existing orthopedic injuries, use of dietary supplements and ongoing medical treatment. Participants were advised to obtain a minimum of 8 h of sleep prior to each testing session and to refrain from strenuous physical activity during the study period. Dietary intake was standardized across both test days, albeit distributed across three meals during the control session and two meals during the fasting session. Caloric intake and macronutrient distribution were set at 40 kcal/kg/day, comprising 20% protein, 30% fat and 50% carbohydrates (2, 8).

### 2.3 Procedures

**Figure 1:**
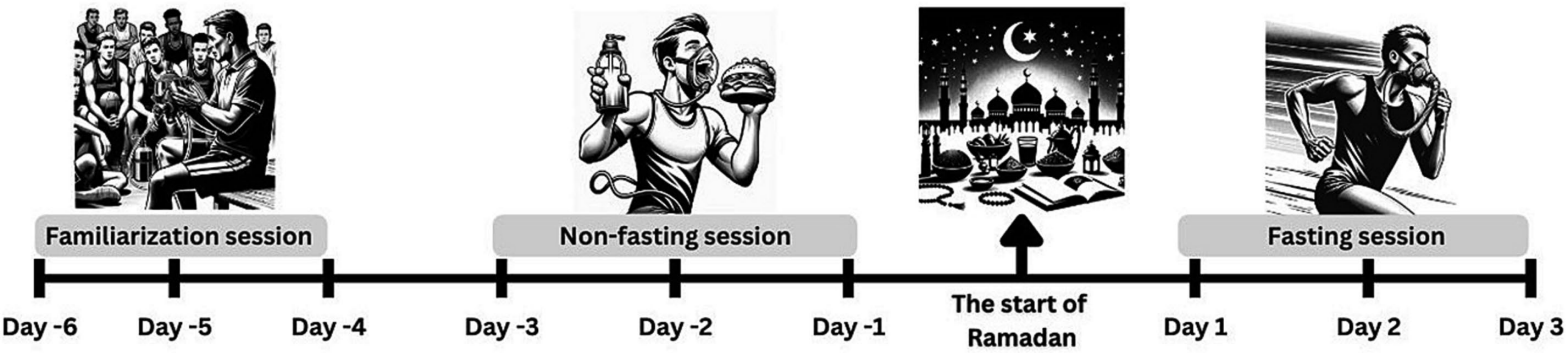
Flowchart of the study design process.

Participants visited the Atatürk University Sports Sciences Implementation and Research Centre on three separate occasions: a familiarization session and two experimental sessions (Figure 1). The familiarization session was scheduled before the onset of Ramadan, during which participants were not fasting. During this session, participants were acclimated to exercising with a respiratory gas mask and a sample lactate measurement was obtained. Subsequently, maximal 20-meter sprint times were recorded twice (with a 3-min interval) and the best (shorter) time was used as the criterion score. Participants were required to achieve at least 95% of this criterion score during the initial sprints of both tests. Failure to do so resulted in test termination and a 5-min rest before reattempting.

The control session (non-fasting) was conducted within the last 3 days before the start of Ramadan. The experimental session (fasting) took place during the first 3 days of Ramadan, ensuring that each participant had a 48–72-h gap between their control and experimental sessions. All tests were administered between 16:00 and 18:00, corresponding to a fasting duration of 12–14 h for the experimental session. Participants executed a repeated sprint protocol consisting of ten 20-meter sprints with 15-s passive recovery intervals. Light-sensitive photocell gates were employed, and athletes were instructed to initiate their sprints following a 3-s countdown indicated by the lighting sequence. Sprints began 50 cm behind the photocell gate. Repeated-sprint performance metrics, including total time and percentage of speed decrement, were calculated based on formulas proposed by Glaister et al. (26).


$$\mathrm{Percentage of speed decrement}=\left[100\times \left(\begin{array}{l} \mathrm{total sprint time}÷\\ \mathrm{ideal sprint time} \end{array}\right)\right]\_100$$

Total sprint time=sumof sprint times fromallsprints

Ideal sprint time=the number of sprints×fastest sprint time

A standardized warm-up protocol was performed before each testing session, consisting of 10 min of jogging and stretching and followed by three 5-meter acceleration drills. Efforts were made to minimize long-duration and high-intensity actions to prevent blood lactate accumulation and fatigue. Testing started 3 min post-warm-up. Anthropometric assessments were conducted using standardized techniques on the initial test day. Height was measured using a portable stadiometer (Holtain Ltd., Crosswell, Crymych, Dyfed, United Kingdom), whereas body mass and fat were evaluated via air displacement plethysmography (Bod Pod, Cosmed, Chicago, IL, United States) calibrated as per manufacturer guidelines. Oxygen consumption (VO_2_) was measured using a portable metabolic gas analyzer (K5, Cosmed, Rome, Italy) during and 15 min post-exercise. Blood lactate concentrations were assessed pre-exercise and at the 1st, 3rd, 5th, 7th and 10th minutes post-exercise, using capillary blood samples from the left-hand fingertip and analyzed with a portable hand analyzer (Lactate Scout +, SensLab GmbH, Germany). Laboratory conditions were maintained at approximately 22°C and 45% humidity throughout all testing sessions.

### 2.4 Calculation of the energy pathway contributions

The contributions of the oxidative, lactic and alactic metabolic pathways were quantified using the PCr-LA-O2 method (27, 28). Oxygen uptake was assessed during exercise and a 15-min post-exercise period using a portable gas exchange system operating in breath-by-breath mode. Prior to each test, the portable metabolic gas analyzer was calibrated in accordance with the manufacturer’s guidelines. Blood lactate concentrations were ascertained from capillary blood samples obtained from the left-hand fingertip using a portable hand analyzer. Blood samples (20 μL) were collected at baseline and at subsequent intervals during the recovery phase (1st, 3rd, 5th, 7th, and 10th minutes) to determine peak lactate levels [BLa_peak_]. The delta lactate concentration [BLa_delta_] was calculated as the difference between BLa_peak_ and baseline values. The oxidative pathway contribution was derived from VO_2_ levels exceeding the standing baseline (4.5 mL·kg^−1^·min^−1^) during the tests. The area under the curve of the actual VO_2_ minus the standing VO_2_ was used to quantify this contribution (29).


(1) $$\begin{array}{l} \mathrm{Oxidative}\\ \mathrm{contribution}\;\left(\mathrm{kj}\right)=\left[\begin{array}{l} \mathrm{actual}\;{\mathrm{VO}}_{2}\;\left(\mathrm{ml}\right)\_\\ \mathrm{standing}\;{\mathrm{VO}}_{2}\;\left(\mathrm{ml}\right) \end{array}\right]/1000\times 20.9 \end{array}$$

The lactic system contribution was calculated from the BLadelta with the di Prampero equivalent (29).


(2) $${\mathrm{BLa}}_{\mathrm{delta}}\;\left(\mathrm{mmol}\right)=\mathrm{Peak}\;\mathrm{BLa}\;\left(\mathrm{mmol}\right)\_\mathrm{Resting}\;\mathrm{BLa}\;\left(\mathrm{mmol}\right)$$

(3) $$\begin{array}{l} \mathrm{Lactic contribution}\;\left(\mathrm{kj}\right)={\mathrm{BLa}}_{\mathrm{delta}}\;\left(\mathrm{mmol}\right)\times \\ \mathrm{body mass}\;\left(\mathrm{kg}\right)\times \\ 3\;\left(\mathrm{ml}\right)]/1000\times 20.9 \end{array}$$

The alactic pathway contribution was determined using bi-exponential curves generated to examine the fast and slow components of excess post-exercise oxygen consumption (EPOC) kinetics utilizing OriginPro software (version 2019b, OriginLab, Northampton, MA, United States). The fast phase of EPOC (actual VO_2_ − slow phase VO_2_) was utilized as the representative of the alactic contribution since ATP-PCr resynthesis occurs in this phase. Additionally, the energy contribution during the breaks was attributed to the alactic pathway considering that rest intervals are predominantly devoted to the repayment of PCr stores. Nonetheless, it should be noted that our calculations operate under the assumption that the post-exercise replenishment of phosphocreatine (PCr) stores is solely mediated by the oxidative pathway. This model does not account for potential minor contributions from the glycolytic system to PCr resynthesis. Additionally, we assume that VO_2_ observed during the fast phase of EPOC is entirely dedicated to the replenishment of PCr stores, thereby neglecting any VO_2_ that may be utilized for the re-binding of myoglobin (29).


(4) $${\mathrm{VO}}_{2\left(t\right)}={\mathrm{VO}}_{\mathrm{2baseline}}+A_{1}\times \left[e^{–(\mathrm{t–td)/}\tau 1}\right]+A_{2}\times \left[e^{–(\mathrm{t–td)/}\tau 2}\right]$$

(5) $${\mathrm{EPOC}}_{\mathrm{fast}}\;{\mathrm{VO}}_{2}=A_{1}\times \tau 1$$

(6) $$\begin{array}{l} \mathrm{ATP}-\mathrm{PCr}\;\mathrm{contribution}\;\left(\mathrm{kj}\right)={\mathrm{EPOC}}_{\mathrm{fast}}\;{\mathrm{VO}}_{2}+\mathrm{number of}\\ \mathrm{breaks}\times \mathrm{integral of}\;\\ {\mathrm{VO}}_{2\left(\mathrm{EPOC}\_\mathrm{15}\_\sec\right)}\times \mathrm{20}\mathrm{.9} \end{array}$$

EPOC_fast_ is alactic pathway contribution calculated from the oxygen kinetic during the fast component of EPOC, VO_2(t)_ is the oxygen uptake at time *t*, VO_2baseline_ is the asymptotic y-value of the curve, A is the amplitude, td is the time delay, *𝜏* is the time constant, VO_2(EPOC_15_sec)_ is the integral value of the curve for the first 15 s (recovery period between the sprints) and 1 and 2 denote the fast and slow components, respectively.

### 2.5 Statistical analysis

Data-processing procedures were conducted using SPSS 21.0 (IBM Corp, Armonk, NY, United States) and OriginPro 2019b software (OriginLab Corp., Northampton, United States). The measures are reported as means and standard deviations. Normality of distribution was verified using the Shapiro–Wilk test. Differences between variables were calculated using the paired sample t-test. In addition, effect sizes were calculated using Cohen’s *d* (30) and were classified according to Hopkins (31). The Hopkins’ classification for interpreting effect sizes is as follows: Effect sizes less than 0.2 are considered trivial, effect sizes between 0.2 and 0.6 are categorized as small, effect sizes between 0.6 and 1.2 are considered moderate, effect sizes between 1.2 and 2.0 are classified as large, and effect sizes greater than 2.0 are considered very large. For comparisons of sprint times within a protocol, a one-way analysis of variance with repeated measurements was used. A two-way analysis of variance (ANOVA) with repeated measures was used to determine the effects of the state (control and fasting), sprints (1, 13, 22, 24, 27, 28, 32–35) and interaction (state×sprints), followed by multiple comparisons. The assumptions of sphericity were assessed by Mauchly’s test. Whenever an assumption was violated, Greenhouse–Geisser correction if epsilon (*ε*) value was <0.75 and Huynh-Feldt correction if ε was >0.75 were applied to the degree of freedom. Statistical tests were deemed to be significant at *p* < 0.05.

## 3 Results

**Figure 2:**
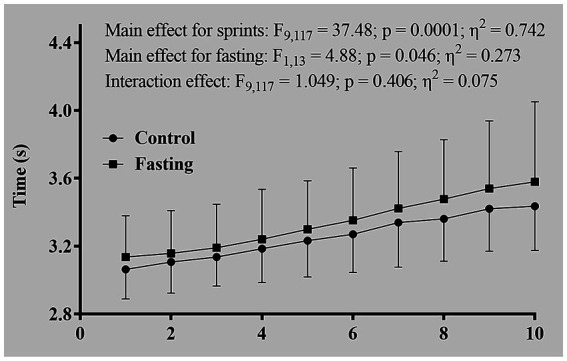
Sprint times of repeated sprints performed during fasting and control sessions.

**Figure 3:**
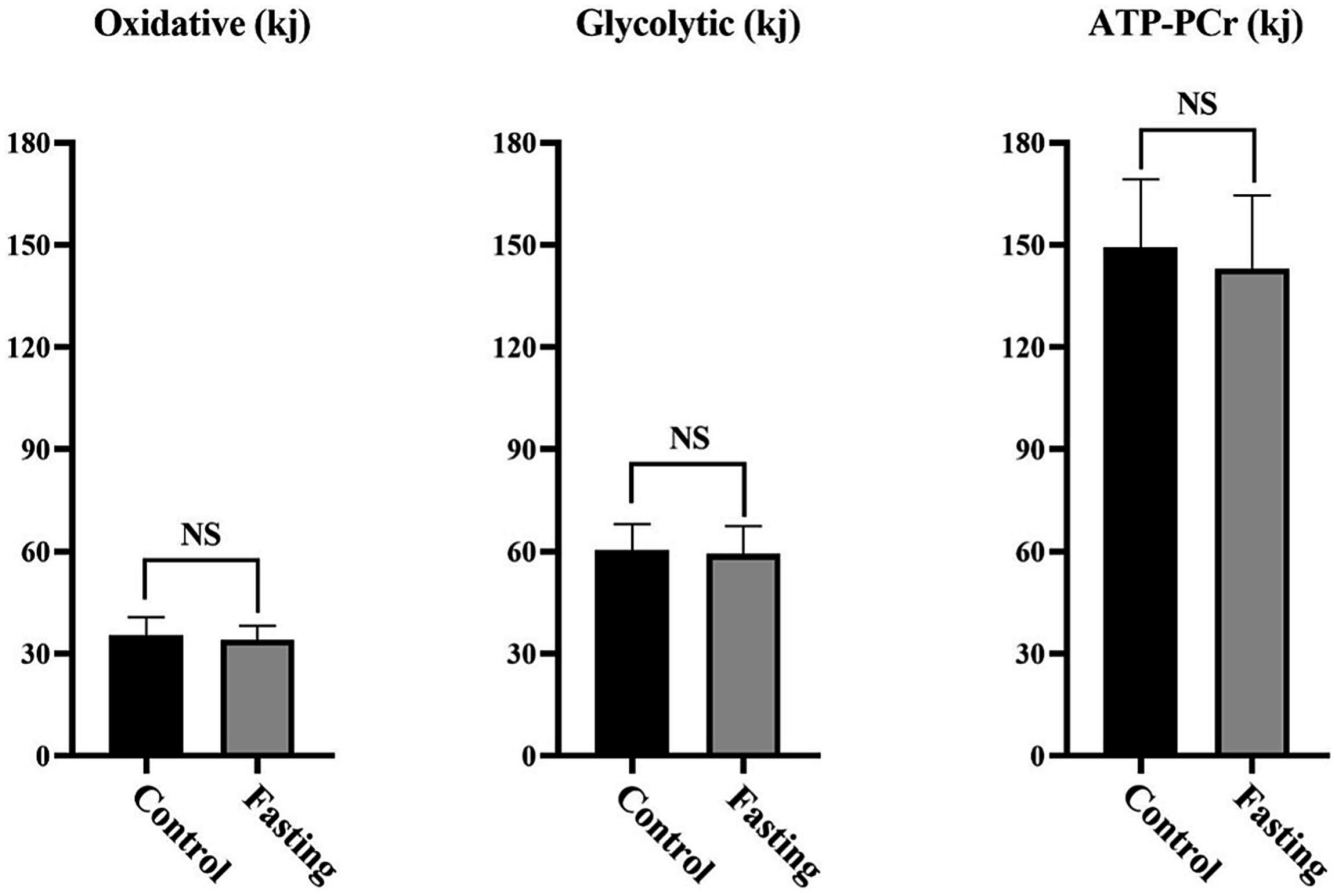
Absolute metabolic energy contribution of the bioenergetic pathways (in kilojoules) during repeated sprints for fasting and control sessions (NS, non-significant).

**Figure 4:**
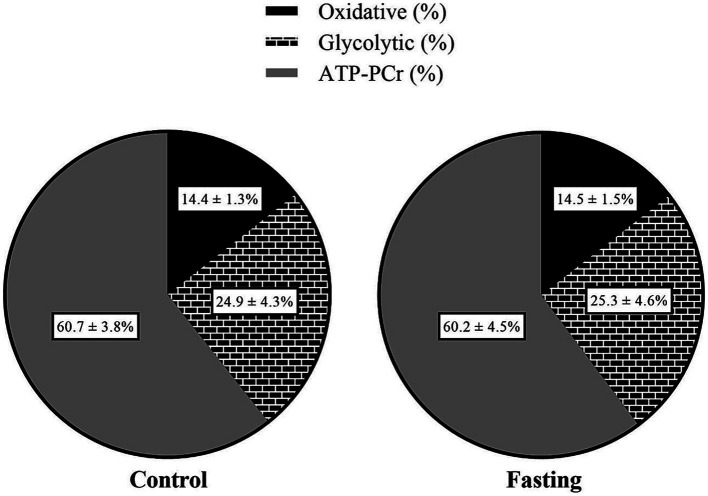
Relative metabolic energy contribution of the respective metabolic pathways (in kilojoules) for fasting and control states.

The worst sprint time during the fasting repeated sprints (RS_fasting_) was significantly higher than that during the non-fasting repeated sprints (RS_control_). However, no differences were recorded in the mean sprint time or the percentage of performance decrement (Table 1). Subsequent analysis two-way ANOVA revealed the main effects for both sprint sequence and nutritional states. Specifically, later sprints exhibited slower times compared to earlier ones and the fasting state was associated with slower times relative to the control state. Importantly, no interaction effect was detected between the nutritional state and the sprint sequence in terms of absolute performance metrics (Figure 2). With respect to metabolic contributions, both in relative and absolute terms, no significant differences were identified in the contributions from the oxidative, lactic and alactic pathways (Table 1; Figures 3, 4).

**Table 1:** Metabolic outputs of repeated sprints performed during fasting and control sessions.

	**RS**_**control**_	**RS**_**fasting**_	**t**	**p**	**ES (d)**
Mean time (s)	3.3 ± 0.2	3.4 ± 0.3	2.144	0.052	0.34
Worst time (s)	3.4 ± 0.3	3.6 ± 0.5	2.180	0.048	0.39
P_dec_ (%)	6.4 ± 2.7	6.6 ± 3.3	0.373	0.715	0.07
Oxidative (kJ)	35.5 ± 5.2	34.2 ± 4.1	1.236	0.238	0.28
Lactic (kJ)	60.4 ± 7.6	59.2 ± 8.3	0.720	0.484	0.15
Alactic (kJ)	149.3 ± 19.9	143 ± 21.5	1.586	0.137	0.30
TEE (kJ)	245.2 ± 21.7	236.5 ± 22	1.759	0.102	0.40
EE per sprint	24.5 ± 2.2	23.7 ± 2.2	1.757	0.106	0.35
Oxidative (%)	14.4 ± 1.3	14.5 ± 1.5	0.798	0.846	0.07
Lactic (%)	24.9 ± 4.3	25.3 ± 4.6	0.551	0.591	0.09
Alactic (%)	60.7 ± 3.8	60.2 ± 4.5	0.596	0.561	0.12

TEE, total metabolic energy expenditure; EE, metabolic energy expenditure; P_dec_, Percentage of performance decrement.

## 4 Discussion

The decline in physical performance during Ramadan has commonly been attributed to factors such as caloric restriction, alterations in macronutrient composition and sleep deprivation. However, the present investigation controlled for these variables and concentrated on shifts in energy metabolism. Our data revealed no statistically significant variations in the contributions of alactic, lactic and oxidative metabolic pathways during RSA in both fasting and non-fasting conditions contrary to our hypothesis. It should be noted, however, that whereas the volume of fluid intake remained constant over the two different conditions, the timing of fluid consumption was markedly different due to the restrictions imposed by the fasting period.

In this study involving athletes positioned in the middle of the training and performance caliber scale, we have identified that bioenergetic activities are similar in both fasting and non-fasting conditions. However, the findings indicate that there may be a tendency for performance outcomes to decline during more demanding exercise tasks while fasting. A recent systematic review by Abaïdia et al. (1) highlighted a critical methodological consideration in interpreting the outcomes of studies on this subject. The review concluded that elite athletes did not experience a significant decline in performance, whereas sub-elite athletes with less frequent training sessions exhibited a more pronounced performance decrement. Given that our study population consisted of trained/developmental-level football players (25), our findings align with this observation suggesting that fasting had a negligible impact on bioenergetic contributions and performance metrics. Nonetheless, the data imply that these effects could become more pronounced under more strenuous and extended exercise protocols. Therefore, it is imperative to consider multiple variables including the athletes’ training level, exercise intensity and duration, fatigue etiology and overall energy metabolism, when evaluating the effects of RIF on physical performance.

This study demonstrates that similar bioenergetic contributions can be achieved during repeated sprints, which predominantly activate the lower body muscle groups, while fasting. It has been established that approximately half of an athlete’s body weight comprises muscle mass, predominantly localized in the lower extremities (36, 37). This translates to an estimated 250–300 grams of glycogen storage in the muscles of athletes engaged in sprint activities. Given that this storage is sufficient to meet the metabolic energy demands of typical sprinting protocols, performance decrements during RIF are likely attributable to factors extrinsic to muscle cell activity, such as dehydration or hypoglycemia. In a standard feeding regimen, elevated blood glucose levels are expected within a few hours post-suhoor (i.e., the meal eaten before dawn during Ramadan). The rate of post-absorptive blood glucose utilization, primarily for hepatic glycogen synthesis and glycolysis, is approximately 2 mg/kg/min, although glucose levels remain comparatively low until the iftar meal (i.e., the meal eaten at sundown during Ramadan; (22, 38)).

During the afternoon phase of RIF, skeletal muscles exhibit metabolic plasticity, transitioning from carbohydrates to lipids as the primary fuel source to conserve hepatic glycogen and maintain blood glucose levels (39, 40). However, high-intensity exercises like sprinting predominantly rely on muscle glycogen. Theoretically, the preservation of muscle glycogen stores until training implies that the fuel requirements during sprints remain consistent with non-fasting conditions. During RSA conducted shortly before the iftar meal, working muscles require high-energy phosphates (ATP and CP) and glucose. Given that blood glucose equilibrium is primarily regulated by the insulin/glucagon balance, hepatic activation and ketone metabolism, it can be inferred that intramuscular glycogen stores are adequate for high-intensity muscle activation. Nonetheless, cognitive impairments associated with low blood glucose levels suggest that even when muscle cell metabolism is regular, disruptions may occur in the neural transmission pathways to the muscle cells.

Alterations in sleep and nutritional patterns during Ramadan induce endocrinological changes (9, 34, 35). Monoamines, particularly serotonin, modulate both sleep and metabolism (35). Prolonged carbohydrate deprivation may prevent tryptophan-dependent drowsiness, as dietary tryptophan is the biochemical precursor of serotonin and plasma tryptophan levels increase following carbohydrate ingestion (35). Additionally, melatonin induces drowsiness through its thermoregulatory effects and direct action on the central nervous system (33, 34). These endocrinological changes are closely related to the mechanisms for maintaining blood glucose and appear to support the use of muscle glycogen as an energy source during sprinting.

Cortisol and testosterone levels typically peak in the early morning (24, 33, 35), optimizing performance in activities requiring maximal effort or significant motor coordination. However, a shift in cortisol secretion patterns during Ramadan has been observed, characterized by reduced morning and increased evening secretion (12, 41). Consistent with these findings, no substantial alterations in respiratory variables were observed during Ramadan (42). Although some studies reported a metabolic slowdown characterized by lower resting afternoon VO_2_ (1, 12, 32), our study suggests that metabolism can adequately respond to increased energy demands during repeated sprints.

In this study, we employed a design that focused on bioenergetic activity during repeated sprints, where both anaerobic and aerobic metabolism are active, while controlling for other variables. Our findings challenge the common assumption that fasting negatively impacts energy metabolism pathways during high-intensity exercise. Notably, the results related to performance were close to the threshold of statistical significance, suggesting that outcomes could vary in more challenging exercise tasks. We implemented a protocol of ten 20-meter repeated sprints with 15-s passive recovery intervals. Such designs have dynamic variables, including increasing the number of sprints, extending sprint distance, or reducing rest time, indicating that results could differ in more demanding exercise tasks. Furthermore, the athlete group in our study, when assessed in terms of training and performance caliber, is situated at the third level of a six-tier scale (2, 12, 24). It’s conceivable that athletes at higher levels could withstand more strenuous exercise tasks even while fasting. Thus, it’s important to remember that many factors need to be considered in such studies. In conclusion, our study highlights the complex nature of bioenergetic performance in athletes during Ramadan fasting and the potential impacts of various factors on this performance. It is clear that more research is needed to fully understand these effects and their implications for athletes’ performance.

## 5 Conclusion

To the best of our knowledge, the present study represents the inaugural investigation of the effects of RIF on repeated sprint performance, with a specific focus on associated bioenergetic pathways. The worst sprint time during the fasting repeated sprints was significantly higher than that during the non-fasting repeated sprints. However, no differences were recorded in the mean sprint time or the percentage of performance decrement. In the context of bioenergetic contributions and total energy expenditure, values were not different between the two conditions. Consequently, while our experimental protocol did not yield unequivocal disparities between the fasting and non-fasting states in terms of performance metrics and metabolic parameters, it intimates that such differences could become more salient in protocols of extended duration and elevated intensity, favoring the non-fasting state.

## 6 Future studies

The study controls for key variables like calorie intake, macronutrient proportions and sleep duration, thereby isolating the effects of fasting on energy metabolism. However, the timing of fluid intake, which is significantly altered during Ramadan, could be a variable worth exploring in future research. Our findings lay the groundwork for future studies to delve deeper into the metabolic markers and energy production mechanisms associated with varying exercise intensities and durations during Ramadan. Understanding these nuances is crucial for athletes and coaches aiming to optimize training and performance during this period. Moreover, the study opens avenues for investigating how religious and cultural practices intersect with sports science, offering a multidisciplinary lens that could enrich both fields. In summary, while our study does not provide definitive answers, it raises pertinent questions and provides a robust framework for future research in exercise physiology, particularly in the context of religious fasting.

## 7 Strengths and limitations

The present study uniquely investigates the effects of fasting on athletic performance from the perspective of bioenergetic contributions. We utilized a comprehensive methodology that allowed us to discern the contributions from all three energy systems—anaerobic alactic, anaerobic lactic, and aerobic—providing a holistic understanding of how fasting impacts different aspects of energy utilization during repeated sprint activities. Furthermore, the use of repeated sprints as the performance metric aligns with the demands of many team sports, enhancing the ecological validity of our findings. Additionally, this study design carefully controlled the timing of data collection to ensure that both fasting and non-fasting sessions occurred within the same week, minimizing potential confounding factors due to time variations. However, it’s important to note some limitations. First, our study had a relatively small sample size of intermediate-level football players, which may limit the generalizability of our findings to elite or sub-elite athletes. Second, the study design focused on the initial days of Ramadan, and different results might be observed in the later stages of fasting. Finally, while we controlled for several variables, there may still be unaccounted factors that could influence the outcomes. Future research with larger and more diverse populations and extended observation periods could provide further insights into the effects of fasting on athletic performance.

## Data availability statement

The raw data supporting the conclusions of this article will be made available by the authors, without undue reservation.

## Ethics statement

The studies involving humans were approved by the Ethics Committee of Atatürk University’s Faculty of Sport Sciences. The studies were conducted in accordance with the local legislation and institutional requirements. The participants provided their written informed consent to participate in this study.

## Author contributions

SÖ: Conceptualization, Data curation, Formal analysis, Investigation, Methodology, Project administration, Resources, Software, Supervision, Validation, Visualization, Writing – original draft, Writing – review & editing. SU: Conceptualization, Data curation, Formal analysis, Investigation, Methodology, Project administration, Resources, Software, Supervision, Validation, Visualization, Writing – original draft, Writing – review & editing. CG: Conceptualization, Data curation, Formal analysis, Investigation, Methodology, Project administration, Resources, Software, Supervision, Validation, Visualization, Writing – original draft, Writing – review & editing. IO: Conceptualization, Data curation, Formal analysis, Investigation, Methodology, Project administration, Resources, Software, Supervision, Validation, Visualization, Writing – original draft, Writing – review & editing. FÖ: Conceptualization, Data curation, Formal analysis, Investigation, Methodology, Project administration, Resources, Software, Supervision, Validation, Visualization, Writing – original draft, Writing – review & editing. HY: Conceptualization, Data curation, Formal analysis, Investigation, Methodology, Project administration, Resources, Software, Supervision, Validation, Visualization, Writing – original draft, Writing – review & editing. NK: Conceptualization, Data curation, Formal analysis, Investigation, Methodology, Project administration, Resources, Software, Supervision, Validation, Visualization, Writing – original draft, Writing – review & editing. FK: Conceptualization, Data curation, Formal analysis, Investigation, Methodology, Project administration, Resources, Software, Supervision, Validation, Visualization, Writing – original draft, Writing – review & editing. SA: Conceptualization, Data curation, Formal analysis, Investigation, Methodology, Project administration, Resources, Software, Supervision, Validation, Visualization, Writing – original draft, Writing – review & editing. İU: Conceptualization, Data curation, Formal analysis, Investigation, Methodology, Project administration, Resources, Software, Supervision, Validation, Visualization, Writing – original draft, Writing – review & editing. LPA: Conceptualization, Data curation, Formal analysis, Investigation, Methodology, Project administration, Resources, Software, Supervision, Validation, Visualization, Writing – original draft, Writing – review & editing.
